# Individual Differences in Automatic Emotion Regulation Affect the Asymmetry of the LPP Component

**DOI:** 10.1371/journal.pone.0088261

**Published:** 2014-02-11

**Authors:** Jing Zhang, Renlai Zhou

**Affiliations:** 1 Beijing Key Lab of Applied Experimental Psychology, School of Psychology, Beijing Normal University, Beijing, China; 2 State Key Laboratory of Cognitive Neuroscience and Learning, Beijing Normal University, Beijing, China; 3 Center for Collaboration and Innovation in Brain and Learning Sciences, Beijing, China; 4 Department of Psychology, Renmin University of China, Beijing, China; University of Akron, United States of America

## Abstract

The main goal of this study was to investigate how automatic emotion regulation altered the hemispheric asymmetry of ERPs elicited by emotion processing. We examined the effect of individual differences in automatic emotion regulation on the late positive potential (LPP) when participants were viewing blocks of positive high arousal, positive low arousal, negative high arousal and negative low arousal pictures from International affect picture system (IAPS). Two participant groups were categorized by the Emotion Regulation-Implicit Association Test which has been used in previous research to identify two groups of participants with automatic emotion control and with automatic emotion express. The main finding was that automatic emotion express group showed a right dominance of the LPP component at posterior electrodes, especially in high arousal conditions. But no right dominance of the LPP component was observed for automatic emotion control group. We also found the group with automatic emotion control showed no differences in the right posterior LPP amplitude between high- and low-arousal emotion conditions, while the participants with automatic emotion express showed larger LPP amplitude in the right posterior in high-arousal conditions compared to low-arousal conditions. This result suggested that AER (Automatic emotion regulation) modulated the hemispheric asymmetry of LPP on posterior electrodes and supported the right hemisphere hypothesis.

## Introduction

The question of how the two separate hemispheres of the brain process emotion has been the focus of many neuropsychological studies employing event-related potentials (ERPs). Some ERPs studies assessed hemispheric asymmetry within a number of tasks, including the determination of emotional content from a picture showing a baby's expression [1], detecting an emotion from sound [2], and memorizing emotional expressions [3]. However, recent studies have documented hemispheric asymmetry in passive viewing tasks, in which participants only view pictures [4]–[12].

Several competing hypotheses have been proposed to explain the neuropsychological mechanisms of ERPs hemispheric asymmetry. The right hemisphere hypothesis suggests that the right hemisphere is dominant over the left for emotional perception and the experience of either positive or negative emotions [4]–[10]. The valence hypothesis refers to anterior hemispheric asymmetry for the production and perception of emotions depending on the valence of that emotion, with the right hemisphere being dominant for negative emotions and the left dominant for positive emotions [13]–[17]. Davidson et al. [18] extended this approach in the approach–withdrawal model suggesting that left sided anterior neural activity is involved in approach related emotions whereas right sided anterior activity is involved in withdrawal related emotions.

ERPs studies focusing on the late positive potential (LPP), a positive slow modulation of the ERPs with a posterior midline scalp distribution and an onset around 500 ms after stimulus presentation, found that the LPP was larger at the right electrodes than at the left electrodes in emotion perception [19]. The LPP component is a sustained positive deflection in the event related potential, which is related to attentional processing and evaluation processing to affective stimuli and that is larger following emotional compared to neutral visual stimuli [20]. A previous study measured the LPP to determine whether the asymmetry of LPP would be modulated by affective evaluative categorization [19]. Participants were asked to evaluatively categorize each food as either positive vs. nonpositive, or nonevaluatively categorize each food as either vegetable vs. nonvegetable. Results revealed that affective evaluative categorizations evoked larger LPP over the right than the left electrodes compared with the LPP evoked by nonevaluative categorizations [19].

Automatic emotion regulation (AER) has been defined as goal-driven change to any aspect of one's emotions without making a conscious decision to do so, without paying attention to the process of regulating one's emotions, and without engaging in deliberate control [21]. Compared with deliberated emotion regulation, which requires attentional resources and is driven by explicit goals, automatic emotion regulation is based on the automatic pursuit of the goal to alter the emotion trajectory [21]. In the studies of emotion hemispheric asymmetry, participants passively viewed pleasant, unpleasant and neutral pictures with no response. Participants with different automatic emotion regulation tendencies would decrease or enhance the feelings of the pictures simultaneously with no explicit goal.

It has been shown that automatic emotion regulation can modulate the arousal of emotion not only by reducing the self-report arousal or maladaptive cardiovascular changes in anger situation, but also by attenuating the amplitude of the LPP component in emotion conditions [22], [23]. Concerning the LPP component, previous studies have documented the amplitude of the LPP decreases as a function of implicit emotion regulation [23]. Hajcak, Moser, & Simons [22] demonstrated that the LPP amplitude decreased when participants were not paying attention to the emotional aspects of the stimuli in the implicit regulation condition. Mocaiber et al. [23] also examined whether the implicit reappraisal strategy could modulate the LPP. Mocaiber et al. [23] invited participants to perform the bar-orientation task under the unpleasant (or neutral) picture distracters in two contexts in which a prior description presented them as taken from either movie scenes (fictitious, provided as a manipulation of implicit reappraisal strategy) or real scenes. They found the LPP amplitude was attenuated under the fictitious context but not in the real context and concluded that responses to affective stimuli could be modulated by an implicit emotion regulation [23]. These findings have led to the view that the LPP is an index of automatic emotion regulation. However, it remains largely unknown, whether automatic emotion regulation affects the asymmetry of the LPP component in emotion conditions. In the present study, we examined whether automatic emotion regulation modulated the hemispheric asymmetry of LPP component, in response to emotion combined with the effects of emotion arousal and valence. We also intended to replicate the findings that emotion arousal would be changed by automatic emotion regulation [21], especially at the level of ERPs.

How to manipulate automatic emotion regulation is an important question. Two kinds of methods have been used in previous studies: one is aimed at seeking the individual differences in automatic emotion regulation by emotion regulation-Implicit Association Test (ER-IAT) [24], resting prefrontal asymmetric activation measurement [25] or emotion regulation questionnaire [26]; the other method attempts to activate different regulation tendency by taking advantage of priming techniques [27], [28]. The present study followed Mauss e. al. [21]'s ER-IAT to separate participants into automatic emotion control and automatic emotion express groups.

In summary, we hypothesized that the asymmetry of the LPP component would be modulated by automatic emotion regulation during the passive viewing of emotion pictures. We expected that the automatic emotion express group, but not the automatic emotion control group, would show right dominance in high arousal conditions. We also expected that automatic emotion control would diminish the difference of right posterior LPP amplitude in high arousal emotion and low arousal emotion.

## Materials and Methods

### Ethics Statement

The protocol of this study was approved by the Institutional Review Board of the State Key Laboratory of Cognitive Neurosciences and Learning of Beijing Normal University. Informed written consent was obtained from each subject before the experiment.

### Participants

Sixty female students (mean age: 23.2±2.53 years, range: 18–28 years) from Beijing Normal University voluntarily participated in the Emotion Regulation-Implicit Association Test (ER-IAT) study. All participants were right-handed. Data from all participants were included in the analysis. Fifteen participants, whose scores in the emotion control category of ER-IAT were in the top 27% of the 60 participants, were recruited into the high-automatic emotion regulation group of the ERP study. Fifteen participants, whose scores in the emotion control category were in the last 27% of the total participants, were recruited into the low-automatic emotion regulation group of the ERP study. One participant was excluded due to technical problems, and four participants did not finish the experiment. All participants had normal or corrected-to-normal vision, reported no medication use at the time of the study and had no history of neurological disorders or drug abuse. Each participant received 15 Chinese yuan for participating in the ER-IAT study and 30 Chinese yuan for participating in the ERP study.

### Stimuli

We adapted the ER-IAT to assess individual differences in the implicit evaluation of emotion regulation [29]. This paradigm has previously been used by Mauss et al. [29] in the assessment of automatic emotion regulation. The present study applied the same paradigm. Twenty items of the ER-IAT were translated into Chinese by a graduate student who majored in psychology. A lecturer who majored in English and psychology translated the twenty Chinese items into English. We changed the Chinese wording if the translation did not match its English equivalent until all twenty Chinese items matched the corresponding English items. We then transferred the Chinese items to the ER-IAT.

The visual stimuli consisted of a picture of a gray square (16×12 cm, RGB, 144, 144, 144) and four sets of 36 emotional pictures drawn from the IAPS, defined as high negative arousal (NH, badly mutilated bodies, threatening animals and events), low negative arousal (NL, gloomy faces, scenes and events), high positive arousal (PH, delicious food, warm groups of people, sweet girls, money and exciting sport scenes, but without sexual pictures) and low positive arousal (PL, cute animals, smiling faces, less exciting sports). The allocation of pictures to these categories had been confirmed in a pilot study, in which 291 Chinese participants (Mean age 21.51 years; SD = 2.45) rated the valence, arousal and dominance of all 704 IAPS pictures on a 9-point Self-Assessment Manikin (SAM; [30], [31]. In the pilot study, participants were instructed to rate what the degrees of the positive or negative feelings are (valence) and how strong the feelings were (arousal) when they watch the picture. The means and standard deviations of the valence and arousal ratings for the four picture sets were as follows: NH: Valence: M = 2.47, SD = 0.66, Arousal: M = 5.80, SD = 0.50; NL, Valence: M = 3.24, SD = 0.95, Arousal: M = 4.79, SD = 0.59; PH, Valence: M = 6.34, SD = 0.77, Arousal: M = 6.09, SD = 0.62; PL, Valence: M = 6.68, SD = 0.83, Arousal: M = 5.40, SD = 0.43. A 2 (Arousal)×2 (Valence) repeated measures ANOVA was conducted on the normative ratings of arousal and valence. The NH and NL blocks differed in arousal (F_(1, 35)_ = 79.35, p<.001) but not in valence (p>.05). The same result also emerged for the PH and PL pictures (F_(1, 35)_ = 40.65, p<.001; p>.05). The NH and PH blocks were significantly different in valence (F_(1, 35)_ = 770.63, p<.001) but not in arousal (p>.05).The same held for the NL and PL pictures (F_(1, 35)_ = 206.06, p<.001; p>.05). The gray square block provided a baseline measure of the state of the participants when viewing a stimulus, which had been applied in the study of Sato and Aoki [32] and the study of Zhang et al. [7]. In our study, the pictures (16×12 cm) were presented on a 17-inch CRT monitor positioned in front of the participant. The distance from the participants' eyes to the screen was 60 cm, with a visual angle of 15° horizontally and 11° vertically to the picture.

### Procedures

#### ER-IAT

This study applied the IAT paradigm, which has been used in the study by Mauss et al. [29]. Participants were told that they should categorize each stimulus word as quickly as possible and with no errors. The task was administered on a 17-inch CRT monitor using the program Inquisit for Windows XP. In Block 1, the participants practiced categorizing items into the positive versus negative categories. In the critical Block 3, the participants finished categorizing 40 items into emotion regulation and positive categories versus emotion expression and negative categories. In Block 2, the participants practiced for 20 items. In Block 5, the participants finished categorizing 40 items into emotion regulation and negative categories versus emotion expression and positive categories. In Block 4, the participants practiced for 20 items.

The participants also completed the Chinese version of the Beck Depression Inventory (BDI) [33] and the State–Trait Anxiety Inventory (STAI) [34] at the very beginning of the experiment.

#### ERPs procedure

Two participant groups took part in the ERPs study. One group was the high-automatic emotion regulation group and the other was the low-automatic emotion regulation group. The experiment was conducted individually in a private suite of a laboratory. Each participant sat in a chair for the placement of 64 Ag-AgCl electrodes on scalp sites identified by the extended International 10–20 System. Following electrode placement, the participant was seated in a comfortable chair in a dimly lit testing room. To reduce muscle artifacts in the EEG signal, the participant was instructed to assume a comfortable position and to avoid movement and unnecessary eye blinks. The presence of an adequate EEG signal was determined by a visual inspection of the raw signal on the monitor. The participant was then asked to view a series of slides that was divided into five blocks based on the picture type (high arousal pleasant, low arousal pleasant, high arousal unpleasant and low arousal unpleasant color pictures and neutral gray squares). Each block included 72 trials. Each trial was comprised of 1,000 ms of picture presentation and a randomized inter-stimulus interval (ISI) of 2,500–3,000 ms.

The participant first completed 72 trials in the neutral block followed by a rest period of 3 min. Thereafter, the participant completed 72 trials in one of the four emotion blocks. Immediately after the completion of 72 trials the participant was asked to complete a self-rating measure of their emotional state during the picture viewing period in terms of arousal (0 = very calm, 9 = highly aroused) and valence (0 = very unpleasant, 9 = very pleasant). The participant then rested for 3 min before the next emotion block and subsequent self-rating survey. Participants completed all emotion blocks, and the order of presentation was randomized.

### EEG Recording

Scalp voltages were recorded by a NeuroSCAN system (according to the 10–20 system) using a 64-channel quick cap with Ag-AgCl electrodes (Neurosoft, Inc. Sterling, USA). Horizontal electro-oculography (HEOG) was recorded bi-polarly from the outer canthi of both eyes, and vertical EOG (VEOG) was recorded from above and below the left eye. All electrodes were referred to the left mastoid with a Fpz ground. Electrode impedance was kept below 5 kΩ. The amplifier bandwidth was .05–100 Hz. EEGs and EOGs were sampled with a digitization rate of 1000 Hz. All EEG and EOG signals were saved on a computer hard disk for offline analysis.

### ER-IAT Analysis

We excluded the trials with latencies greater than 10,000 ms. Response latencies were log-transformed and aggregated separately for trials with compatible (emotion regulation and positive vs. emotion expression and negative) and incompatible (emotion regulation and negative vs. emotion expression and positive) response assignments. Then, we calculated the SDs across the practice and test trials. The means of the latencies of practice and test trials were divided by the resulting SDs. Finally, we subtracted the means of Block 3 from the means of Block 5. The data of all 60 participants were analyzed. Higher scores indicate more positive implicit evaluation of emotion regulation relative to emotion expression.

### ERP Analysis

The EEG data of four participants displaying persistent muscle artifacts or frequent blinking were excluded from analysis. Offline ocular artifacts were corrected with Neuro-Scan EDIT (Version 4.3.1). The trigger threshold for ocular artifacts was set to 10%. The minimum number of sweeps that were required to construct an averaged VEOG artifact was 2. The duration of the average artifact was 400 ms. After correcting for ocular artifacts, the continuous EEG data were segmented into epochs from 100 ms pre-stimulus to 1,000 ms post-stimulus. The 100 ms pre-stimulus epoch served as the baseline. EEGs were de-trended and baseline-corrected. Epochs exceeding the range of −50 to 50 µV at any channel except HEOG and VEOG were rejected as artifacts. The remaining trials were averaged for each operation separately for each participant. The averaged waveform was filtered with a low pass of 40 Hz (zero phase, 12 dB/octave). The overall average was obtained by averaging each participant's averages separately for each block.

The data were divided into epochs of 1,100 ms, starting from 100 ms before stimulus onset and ending 1,000 ms after stimulus onset. Epochs exceeding the range of −70 to 70 µV at any channel except HEOG and VEOG were rejected as artifacts. Trials with eye blinks, lateral eye movements or overt responses were excluded (these trials represented no more than 5% of the number of trials in one block). The mean ERP amplitudes for all participants were calculated for epochs of the neutral and the four emotion blocks. To obtain the difference epoch, the mean amplitude of the epoch in which pictures were viewed for each emotion block was reduced by those from viewing gray pictures. The different epochs were further calculated for the LPP component for the time window from 500 to 800 ms at four different electrode clusters depicted (left-frontal electrodes: FP1, AF3, AF7, F3, F5 and F7; right-frontal electrodes: FP2, AF4, AF8, F4, F6 and F8; left-parietal electrodes: P3, P5, P7, PO3, PO5 and PO7; right parietal electrodes: P4, P6, P8, PO4, PO6 and PO8). The significance level was set at .05 (two-tailed). According to Heller [35] and Keil et al. [36], the frontal and parietal lobes contribute to different asymmetry patterns, therefore the amplitude analysis of the ERPs in this study was conducted at frontal and parietal electrodes independently. No analysis was conducted for time latency, as peak latency is typically not influenced by affective stimulus value [37].

## Results

### Behavioral results of ER-IAT

The mean of BDI scores before ER-IAT was 7.00 (SD = 4.19). The mean of STAI-T scores before ER-IAT was 41.50 (SD = 6.71). The score of 60 participants were 1124.15 ms (SD = 566.05) in compatible response assignments and 1210.94 ms (SD = 584.97) in incompatible response assignments. Fifteen participants, whose scores in the emotion control category of ER-IAT were in the first 27% of the 60 participants, were recruited into the high-automatic emotion regulation group. Fifteen participants, whose scores in the emotion control category were in the last 27% of the total participants, were recruited into the low-automatic emotion regulation group. Finally, 24 participants (mean age = 23.2, SD = 2.5) took part in the ERP experiment. Table 1 shows the means and SD of the two groups. An independent t test showed that these two groups were significantly different in emotion control score t_(2,24)_ = 10.811 (p<.001).

**Table 1:** The scores of emotion control category of ER-IAT of automatic emotion control group and automatic emotion express group.

	The scores of emotion control category of ER-IAT	
	M	SD
Automatic emotion control group	0.3	0.15
Automatic emotion express group	−0.18	0.11

### ERP results at anterior lobe electrodes

**Figure 1:**
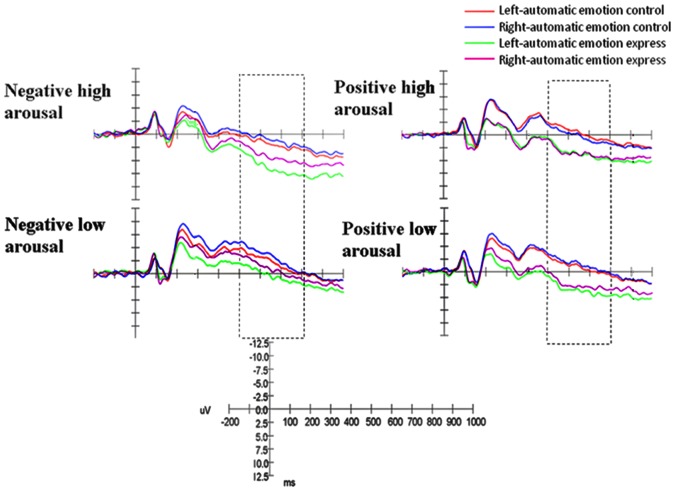
ERPs for automatic emotion control and automatic emotion express groups during four emotion blocks at anterior electrodes. Left = left electrodes. Right = right electrodes.

The ANOVA was performed on the LPP component at anterior lobe electrodes with one between-subject factor: automatic emotion regulation (control group and express group), and with three within-subject factors: arousal (high arousal and low arousal), valence (positive and negative) and asymmetry (left and right). The results revealed significant main effect of arousal on LPP component (F_(1, 23)_ = 23.240, p<.001, η_p_^2^ = .503) with larger mean amplitude in high-arousal emotion than for low-arousal emotion, and significant valence×laterality (F_(1, 23)_ = 4.475, p<.05, η_p_^2^ = .163) on LPP component. Simple effect analysis revealed no significant difference (p>.05). Another significant valence×arousal interaction (F_(1, 23)_ = 14.981, p<.001, η_p_^2^ = .394) was also found on the LPP component. Simple effect analysis showed that larger LPP amplitude in high negative arousal emotion than that in low negative arousal emotion (p<.05), and larger LPP amplitude in low negative arousal emotion than that in low positive arousal emotion (p<.05). No other main effects or interaction effects were found (Fig. 1).

### ERP results at posterior lobe electrodes

**Figure 2:**
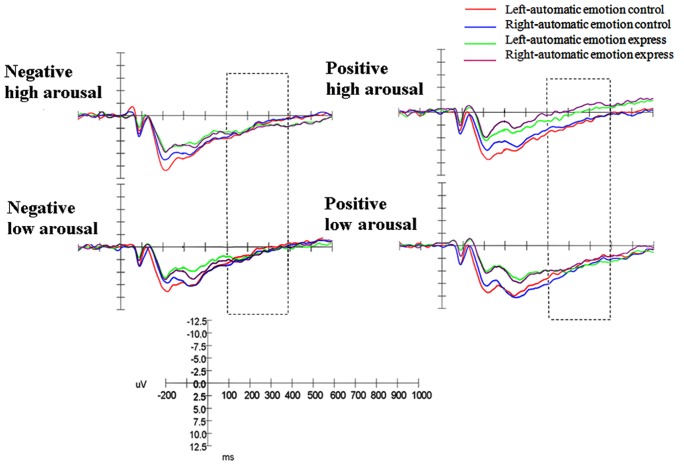
ERPs for automatic emotion control and automatic emotion express groups during four emotion blocks at posterior electrodes. Left = left electrodes. Right = right electrodes.

The ANOVA was performed on the LPP component at posterior lobe electrodes with one between-subject factor: automatic emotion regulation (control group and express group), and with three within-subject factors: arousal (high arousal and low arousal), valence (positive and negative) and asymmetry (left and right). Statistically significant interactions were shown on the LPP component: valence×arousal (F_(1, 23)_ = 49.145, p<.001, η_p_^2^ = .681) and arousal×laterality×group (F_(1, 23)_ = 4.639, p<.05, η_p_^2^ = .189). Simple effect analysis revealed that larger LPP amplitude appeared in negative high arousal emotion than in positive high arousal emotion (p<.001). The three-way simple effect analysis revealed that automatic emotion express group had larger LPP amplitude in the right posterior lobe than the left posterior lobe (p<.05), but automatic emotion control group showed no significant difference (p>.05) in high arousal emotion conditions. The result also showed that the automatic emotion express group showed larger LPP amplitudes at right electrodes in high arousal emotion than that in low arousal emotion (p<.05), but no such difference was observed in the automatic emotion control group (p>.05) (Fig. 2).

## Discussion

The present study examined whether individual difference in automatic emotion regulation (AER) affected emotion-related hemispheric asymmetry for LPP component, with the interaction between valence and arousal. Importantly, we observed a right dominance of LPP component at posterior electrodes in high arousal conditions for automatic emotion express group, but no similar difference was observed for automatic emotion control group. This result suggested that AER modulated the hemispheric asymmetry of LPP on posterior electrodes and supported the right hemisphere hypothesis. We also observed an attenuated difference in the LPP amplitude at right posterior electrodes between high arousal and low arousal emotion for automatic emotion control group compared with automatic emotion express group. This suggested that a declined LPP component on right posterior electrodes would be an indicator of automatic emotion control, especially when compared to automatic emotion express condition.

This result partly supported our hypothesis that AER modulated the right dominance of the mean amplitude of the LPP in posterior electrodes, and also documented the right hemisphere hypothesis, which proposed a dominance of the right lobes in emotion processing. More importantly, our result provided an indication that the right dominance of the LPP component in posterior electrodes happened just for automatic emotion express participants, but not for automatic emotion control participants.

The findings also showed an attenuated difference for automatic emotion control participants in the LPP amplitude at right posterior electrodes between high arousal and low arousal emotion, compared with automatic emotion express participants. Previous researches suggested that the LPP reflected the evaluation of emotion and could be an index of emotion regulation; when emotion regulation happens, the amplitude of LPP would be diminished. For example, Hajcak and Nieuwenhuis found cognitive re-appraisal strategy diminished the amplitude of the LPP and reduced self-reporting negative feelings [38]. The present finding indicated that automatic emotion regulation, including emotion control and emotion express, would regulate the amplitudes of the LPP component in emotion conditions and induce emotion change with no deliberated manipulation.

It would be possible to say that our findings supported the hypothesis that the LPP component would be an index of automatic emotion regulation on the ERPs level. However, some shortcomings in the present study limited our understanding of the LPP component as the index of automatic emotion regulation. Firstly, it is important that deliberated conditions could be manipulated compared with automatic conditions. Previous studies have documented the LPP component as an index of emotion regulation [39]. Thus, future studies should examine the difference of the LPP component in automatic emotion regulation and deliberated emotion regulation. Secondly, although ER-IAT is an effective way to assign participants to automatic emotion control group and automatic emotion express group, as has been evidenced by our findings and Mauss et al.'s study, future studies will be needed to directly speak to the usage of automatic emotion regulation in the experiment [29].

This study provided some indication on the ERPs level that ER-IAT could predict the tendency of automatic emotion regulation, including automatic emotion control or automatic emotion express. This was consistent with Mauss et al.'s study, in which they examined the relationship of automatic emotion regulation and the feeling of anger, cardiac activity and skin conductivity level and found that a higher score in ER-IAT was related to lower levels of physiological index and self-reported feelings of anger [29].

## Conclusion

The present findings provide further evidence that automatic emotion regulation affects the asymmetry of the LPP component when participants view affective pictures passively. Our results showed that a right dominance of the LPP component at posterior electrodes appeared for automatic emotion express participants, but not for automatic emotion control ones. In this way, our study provides further support to the right hemisphere hypothesis.
